# Radiomics of the Paranasal Sinuses: A Systematic Review of Computer-Assisted Techniques to Assess Computed Tomography Radiological Data

**DOI:** 10.1177/19458924241304082

**Published:** 2024-12-16

**Authors:** Rhea Darbari Kaul, Peta-Lee Sacks, Cedric Thiel, Janet Rimmer, Larry Kalish, Raewyn Gay Campbell, Raymond Sacks, Antonio Di Ieva, Richard John Harvey

**Affiliations:** 1 Rhinology and Skull Base Research Group, Applied Medical Research Centre, 7800 University of New South Wales, Sydney, Australia; 2 Computational NeuroSurgery (CNS) Lab, Macquarie Medical School, Faculty of Medicine, Human and Health Sciences, 150782 Macquarie University, Sydney, Australia; 3 Macquarie Medical School, 430860 Faculty of Medicine, Health and Human Sciences, Macquarie University, Sydney, Australia; 4 Woolcock Institute, University of Sydney, Sydney, Australia; 5 Faculty of Medicine, Notre Dame University, Sydney, Australia; 6 Department of Otolaryngology, Head and Neck Surgery, 2659 Concord General Hospital, University of Sydney, Sydney, Australia; 7 Faculty of Medicine, University of Sydney, Sydney, Australia; 8 Department of Otolaryngology Head and Neck Surgery, 2205 Royal Prince Alfred Hospital, Sydney, Australia; 9 School of Clinical Medicine, St Vincent's Healthcare Clinical Campus, Faculty of Medicine and Health, UNSW, Sydney, Australia

**Keywords:** artificial intelligence, diagnostics, prognostics, radiomics, rhinology, machine learning, sinus, computed tomography, chronic rhinosinusitis, nasopharyngeal carcinoma

## Abstract

**Background:**

Radiomics is a quantitative approach to medical imaging, aimed to extract features into large datasets. By using artificial intelligence (AI) methodologies, large radiomic data can be analysed and translated into meaningful clinical applications. In rhinology, there is heavy reliance on computed tomography (CT) imaging of the paranasal sinus for diagnostics and assessment of treatment outcomes. Currently, there is an emergence of literature detailing radiomics use in rhinology.

**Objective:**

This systematic review aims to assess the current techniques used to analyze radiomic data from paranasal sinus CT imaging.

**Methods:**

A systematic search was performed using Ovid MEDLINE and EMBASE databases from January 1, 2019 until March 16, 2024 using the Preferred Reporting Items for Systematic Reviews and Meta-analyses (PRISMA) checklist and Cochrane Library Systematic Reviews for Diagnostic and Prognostic Studies. The QUADAS-2 and PROBAST tools were utilized to assess risk of bias.

**Results:**

Our search generated 1456 articles with 10 articles meeting eligibility criteria. Articles were divided into 2 categories, diagnostic (n = 7) and prognostic studies (n = 3). The number of radiomic features extracted ranged 4 to 1409, with analysis including non-AI-based statistical analyses (n = 3) or machine learning algorithms (n = 7). The diagnostic or prognostic utility of radiomics analyses were rated as excellent (n = 3), very good (n = 2), good (n = 2), or not reported (n = 3) based upon area under the curve receiver operating characteristic (AUC-ROC) or accuracy. The average radiomics quality score was 36.95%.

**Conclusion:**

Radiomics is an evolving field which can augment our understanding of rhinology diseases, however there are currently only minimal quality studies with limited clinical utility.

## Introduction

The role of medical imaging is currently also swiftly evolving from a diagnostic tool to assessed by human beings medical experts to a computational and data-analysis by automated computerized methods.^1,2^ Techniques such as “radiomics” which allow the extraction of quantitative features from medical imaging are now being used to produce large datasets for clinical problem solving.^3^ Radiomics can capture tissue and lesion characteristics and features such as contrast, heterogeneity, and shape.^4^ Broadly speaking, radiomic features can be divided into multiple categories based upon types of features.^4–7^ First-order features including histogram-based properties, for example, gray-level mean, median, and range for individual voxels.^4–7^ Second-order features including textural features, for example, statistical interrelationships between neighboring voxels related to intralesion heterogeneity.^4–7^ Higher order features are related to patterns analysis through statistical methods or mathematical transformations.^4–7^ Artificial intelligence (AI) techniques offer analysis of this data and has already been established in some areas of medical imaging to potentially augment future radiologists’ practices.^2^ Following radiomics-based data extraction, large datasets can be analyzed through conventional statistical methods and/or AI methodologies (eg, machine learning algorithms).^5,8–10^ In medicine, there has been a rapid expansion in the use of radiomics and AI methods in consideration of their ability to identify tumor genotypes and imaging biomarkers with clinical relevance.^5,7–10^ In the field of rhinology, which heavily relies on the use of computed tomography (CT) imaging of the paranasal sinus for diagnostics and assessment of treatment outcomes, there are few applications of radiomic analysis to common conditions.^11^ This systematic review aims to assess the current techniques used to analyze radiomic data from CT images of the paranasal sinuses in rhinology.

## Methods

A systematic review of the literature on the assessment of radiomics in CT paranasal sinus imaging was conducted. The Preferred Reporting Items for Systematic Reviews and Meta-analyses 2020 Checklist (PRISMA 2020) and Cochrane Library Systematic Reviews for Diagnostic and Prognostic Studies were referenced for this review.^11–13^

### Eligibility Criteria

Eligible studies described their approach to radiomics in paranasal sinus CT imaging for feature and pattern recognition in the field of rhinology. Articles included adults 18 years of age and above and were published in the past 5 years (from 2019 to current) to capture up-to-date literature in this rapidly evolving field of research. Gray literature including case reports, conference, and poster abstracts were excluded. Systematic reviews or literature reviews were utilized in bibliographic searching for further relevant articles for inclusion. Articles were excluded if they did not utilize radiomics and only utilized deep learning/machine learning. Articles which utilized other imaging modalities, that is, positron emission tomography (PET), magnetic reference imaging (MRI), X-ray or detailed regions not included in a CT paranasal sinus scan were excluded. Articles which were used for forensics, dental, or radiotherapy treatment were excluded to focus on papers relevant to rhinology.

### Information Sources

A systematic electronic search was performed for relevant studies using the Ovid MEDLINE and EMBASE databases from January 1, 2019 until the March 16, 2024 using a defined search strategy.

### Search Strategy

Radiomics terms including “RADIOMIC” and “textur* adj2 analys*”, Rhinology terms including “NOSE”, “Rhin*” and investigation terms including “CT” and “Comput* adj2 Tomograph*” were combined using the Boolean operators (AND and OR) for the search strategy (see Table 1). A manual bibliographic screen from the relevant studies and systematic reviews was performed to search for additional relevant articles.

**Table 1.** Search Strategy.

Radiomic/imaging terms	AND	Imaging terms	AND	Rhinology terms
1. RADIOMICS		17. CT		22. Rhin*
2. Textur* adj2 analys*		18. CAT adj2 scan		23. Sinonas*
3. Textur* adj2 feature*		19. Comput* adj2 Tomograph*		24. Meatal*
4. Feature adj2 selection		20. COMPUTER ASSISTED TOMOGRAPHY		25. Meatus*
5. Feature adj2 extraction		21. Cone adj2 beam*		26. Nasal*
6. Imaging adj2 analys*				27. Naso*
7. Image adj2 segement*				28. NOSE
8. Automat*				29. Nose*
9. COMPUTER ANALYSIS				30. (Ethmoid* OR Frontal* OR Maxil* OR Spheno*) adj2 sinus*
10. COMPUTER VISION				31. AERD
11. Computation*				32. ASPIRIN-EXACERBATED RESPIRATORY DISEASE
12. Pixel*				33. AFRS
13. Voxel*				34. AFS
14. Hounsfield*				35. allergic adj2 fung*
15. Gray adj2 level*				36. CCAD
16. Grey adj2 level*				37. Central compartment*
				38. RHINOSINUSITIS
				39. ACUTE RHINOSINUSITIS
				40. CHRONIC RHINOSINUSITIS
				41. SINUSITIS
				42. CRS*
				43. Chronic adj2 rhinosinusitis
				44. eCRS*
				45. fung* adj2 ball
				46. fung* adj2 mycetoma
				47. silent adj2 sinus*
				48. nasal adj2 polyp*
				49. NOSE POLYP
				50. Invert* adj2 papilloma*
		51. 1-16				
		52. 17-21				
		53. 22-50				
		54. 51 AND 52 AND 53				
		55. Limit 54 to 2019-currrent				

Table of terms used in MEDLINE (2017–2024) and EMBASE (2017–2024) databases. Search performed on March 16, 2024.

### Selection Process

OVID was utilized to search the MEDLINE and EMBASE databases. Studies were exported to Rayyan (Qatar Computing Research Institute, Qatar), an online review website.^14^ Duplicate studies were automatically removed using the Rayyan duplicate removal function, and also manually reviewed by the author. Study selection was performed by the author (RDK). Any uncertainties were resolved by 2 independent authors (RJH and LK). Studies were screened in 3 phases: first by title, then by abstract, and finally by full text. Articles that met the eligibility criteria were included for data extraction.

### Data Extraction

Data extracted from individual studies were recorded by one author (RDK) in Microsoft Excel. Data fields collected included:

- Study details: authors, year of publication, study design, and country of publication;
- Category of research, that is, diagnostic or prognostic studies;
- Pathology of interest;
- Participant details: number of participants, and the distribution with or without disease of interest;
- Radiomics utilized including software used, number of features, feature selection methods;
- Analysis methods utilized including classification models or validation models; and
- Outcome measures including diagnostic or prognostic performance as applicable, that is, area under the curve receiver operating characteristic (AUC-ROC), sensitivity, specificity, positive predictive value (PPV), negative predictive value (NPV), and accuracy for the diagnostic studies, and AUC-ROC, C statistic, calibration slope, and calibration intercept for the prognostic studies.

*Study risk of bias assessment:* The QUADAS-2 tool for diagnostic studies and the PROBAST tool for prognostic studies were used as per the Cochrane systematic reviews for diagnostic studies and prognostic studies handbook.^15,16^

*Level of evidence:* The level of evidence for each study was recorded as per the Oxford Centre for Evidence-Based Medicine 2011 Levels of Evidence table.^17^

*Radiomics quality assessment:* The radiomics quality scores (RQS) were calculated for the included studies, as a published and previously validated score used to assess the methodology, analysis, and reporting of radiomic studies.^1,18^ This questionnaire comprises of 6 domains (image protocol, radiomics features extraction, data analysis and statistics, model validation, clinical validity, and open science) with 16 items altogether rated up to a total of 36 points, with 36 points (100%) indicating optimal quality. Currently, there are no available score ranges to characterize a rating of quality in the RQS, however we note that similar radiomics quality scoring tools such as METRICS which are yet to be validated have utilized; 0 ≤ score < 20%, “very low”; 20 ≤ score < 40%, “low”; 40 ≤ score < 60%, “moderate”; 60 ≤ score < 80%, “good”; and 80 ≤ score ≤ 100%, “excellent” quality.^19^ One author (RDK) reported the RQS for each study using the online tool.^18^

**Figure 1.**
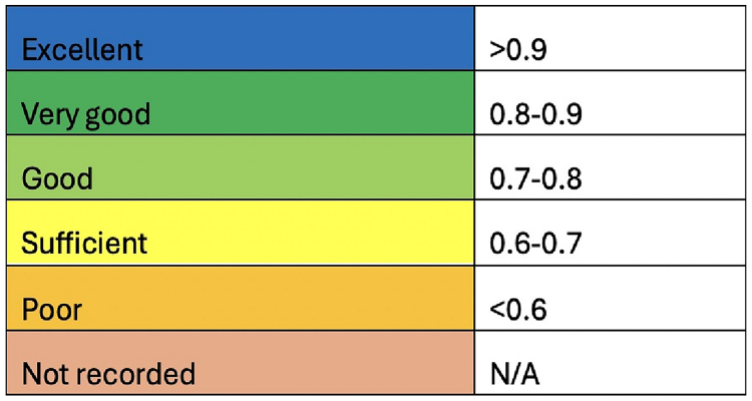
Diagnostic/prognostic utility score. Scoring used for included studies measured with either area under the curve receiver operating characteristic (AUC-ROC) or accuracy.

*Effect measures:* To assess diagnostic and prognostic accuracy, studies reported varying metrics. The most reported metrics for diagnostic studies were the AUC-ROC and accuracy, while the most common for prognostic studies were AUC-ROC and the C statistic. This systematic review used a diagnostic and prognostic utility scoring based on AUC-ROC or accuracy (see Figure 1).^20^ Due to the heterogeneity of the studies included, a meta-analysis was not performed.

## Results

### Study Selection

**Figure 2.**
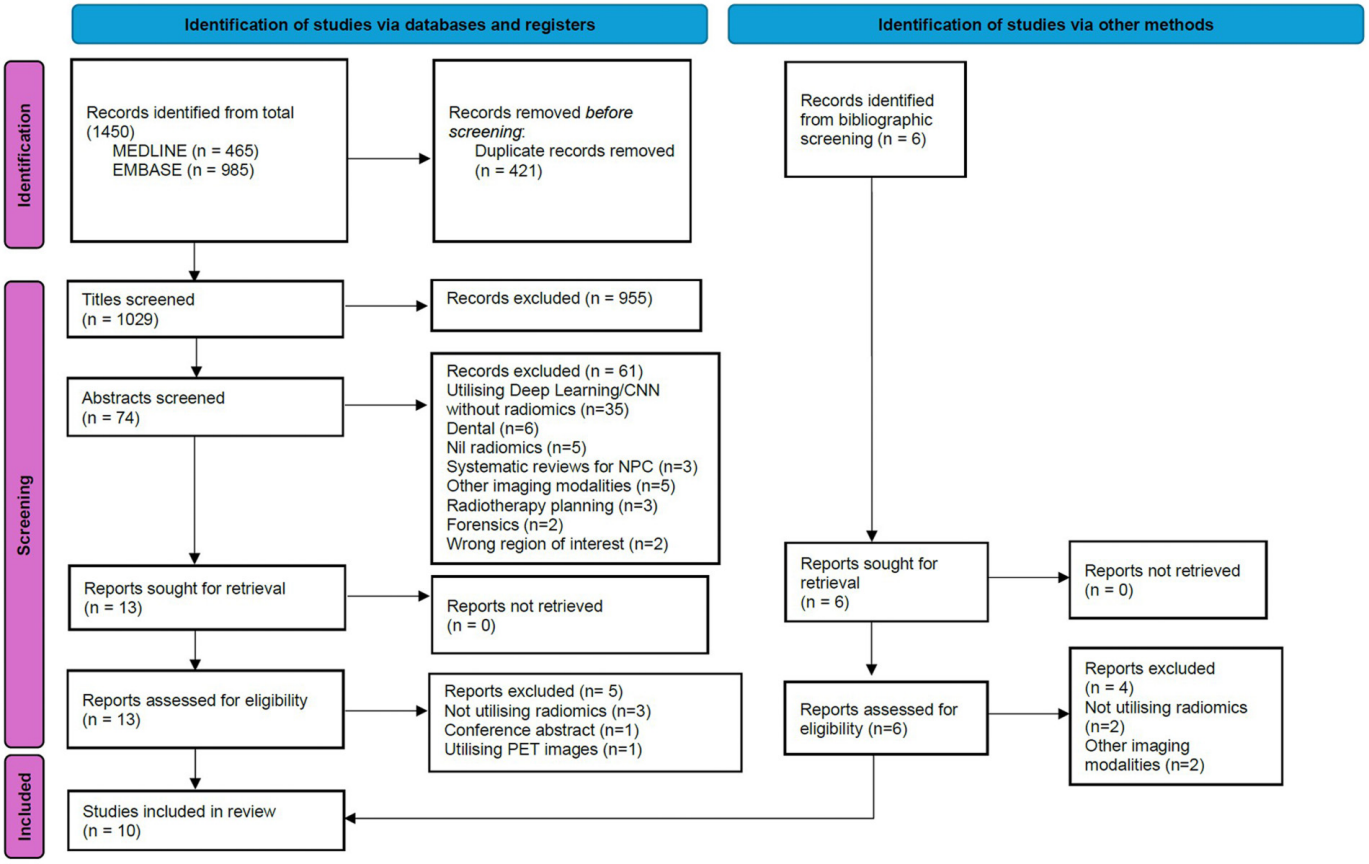
Preferred reporting items for systematic reviews and meta-analyses (PRISMA) flow chart. Study selection process for this review.

The search strategy on MEDLINE and EMBASE resulted in 1450 studies for review. After removal of duplicates, the total was 1029 studies. Following title screening, there were 74 studies and post abstract screening, there were 13 studies identified for full text review. An additional 6 studies were included in the search through bibliographic screening. This resulted in a total of 19 studies for full text review. Of the 19 studies, 5 did not utilize radiomics, 1 was a conference abstract only, and 3 utilized other imaging modailities. These articles were excluded, leaving 10 included studies (see Figure 2).

### Study Characteristics

The 10 included studies were categorized into 2 broad areas; diagnostic or prognostic studies (see Table 2). Studies were all recent, with publication in 2019 (n = 1)^21^, 2021 (n = 2)^22,23^, 2022 (n = 4)^24–27^, and 2023 (n = 3)^28–30^. Most included studies were diagnostic cross-sectional studies (n = 6)^22,24,26,28–30^, along with case series (n = 2)^21,27^ and retrospective cohort studies (n = 2)^23,25^. The number of radiomic features utilized varied from 4 to 1409, with 2 studies using feature selection methods.^27,29^ Machine learning-based analysis was utilized in 7 studies^21–23,25,27,29,30^, with the methods including Least Absolute Shrinkage and Selection Operator (LASSO) logistic regression (n = 2)^23,30^, logistic regression alone (n = 1)^25^, LASSO with ElasticNet (n = 1)^22^, unsupervised clustering (n = 1)^21^, and a combination of classification models (n = 2)^27,29^. Cross validation (CV) models were used in 5 studies^22,23,25,29,30^, with 10-fold CV most frequently used in 3 studies^23,29,30^.

**Table 2.** Table of Characteristics of Included Studies.

Category	Author	Year	Country	Design	Pathology	Subjects	Split of pathologies	Software used	No. of radiomic features	Feature selection methods	Use of AI methods	Classification model	CV model
Diagnostics	Baracnovic et al^22^	2022	Serbia	Cross-sectional	Allergic Fungal rhinosinusitis versus non-allergic Fungal rhinosinusitis	37	24:13:00	Image J	4	N/A	No	N/A	N/A
Diagnostics	Costa et al^23^	2023	Brazil	Cross-sectional	Odontogenic versus Non-odontogenic maxillary sinusitis	40	20:20	MaZda	11	N/A	No	N/A	N/A
Diagnostics	Guo et al^24^	2023	China	Cross-sectional	Nasal polyps versus Inverting papilloma	296	144: 152	Python	1288	Boruta, Random Forest, Correlation coefficient	Yes	SVM, Naïve Bayes, XGBoost	10 fold CV
Diagnostics	Ito et al^25^	2022	Japan	Cross-sectional	Odontogenic versus Non-odontogenic maxillary sinusitis	36	18:18	MATLAB	45	N/A	No	N/A	N/A
Diagnostics	Liang et al^27^	2019	Singapore	Case series	Nasopharyngeal carcinoma	100	N/A	PyRadiomics, Moddicom	70	N/A	Yes	Unsupervised clustering	Nil
Diagnostics	Ogawa et al^28^	2021	Japan	Cross-sectional	Olfactory neuroblastoma versus Sinonasal squamous cell carcinoma	43	17:26	SlicerRadiomics	42	N/A	Yes	LASSO, Elastic Net	Leave one out cross validation
Diagnostics	Zhu et al^29^	2023	China	Cross-sectional	Eosinophilic CRS with nasal polyps versus Non-eosinophilic CRS with nasal polyps	431	N/A	PyRadiomics	10	N/A	Yes	LASSO-LR	10 fold CV
Prognostication	Intarak et al^30^	2022	Thailand	Retrospective cohort	Nasopharyngeal carcinoma	197	N/A	PyRadiomics	842	N/A	Yes	Logistic regression	5 fold CV
Prognostication	Li et al^26^	2022	China	Case series	Chronic rhinosinusitis	91	N/A	MaZda	275	Fisher coefficient, classification error probability, combined average correlation coefficient and mutual information	Yes	Original data analysis, principal component analysis, linear classification analysis, nonlinear classification analysis	Nil
Prognostication	Yan et al^31^	2021	China	Retrospective cohort	Nasopharyngeal carcinoma	311	N/A	AccuContour	1409	N/A	Yes	LASSO-LR	10 fold CV

Includes details of all diagnostic and prognostic radiomic studies of computed tomography paranasal sinus imaging and highlights use of AI methods such as classification and CV models.

Abbreviations: AI, artificial intelligence; CV, cross validation.

### Risk of Bias

For the diagnostic studies, bias by the QUADAS-2 tool was generally low (Table 3) and very low for the prognostic studies, as reported by PROBAST tool (Table 4).

**Table 3.** Risk of Bias for Diagnostic Studies.

Author	Year	Risk of bias				Concerns of applicability		
		Patient selection	Index test	Reference standard	Flow and timing	Patient selection	Index test	Reference standard
Baracnovic et al	2022	Unclear	Unclear	Low	High	Low	Low	Low
Costa et al	2023	Low	Unclear	Low	High	Low	Low	Low
Guo et al	2023	Unclear	Low	Low	Unclear	High	Low	Low
Ito et al	2022	High	High	Unclear	Unclear	Low	Low	Low
Liang et al	2019	Unclear	High	Low	Low	Low	Low	Low
Ogawa et al	2021	Unclear	Unclear	Low	Unclear	Low	Low	Low
Zhu et al	2023	Unclear	Unclear	Low	Low	Low	Low	Unclear

Risk of bias assessment using the Quality Assessment of Diagnostic Accuracy Studies (QUADAS) 2 Tool where red denotes high risk, green low risk, and yellow unclear risk.

**Table 4.** Risk of Bias for Prognostic Studies.

Author	Year	Risk of bias				Concerns of applicability			Overall assessment	
		Participants	Predictors	Outcome	Analysis	Participants	Predictors	Outcome	RoB	Applicability
Intarak et al	2022	Low	Low	Unclear	Low	Low	Low	Unclear	Low	Unclear
Li et al	2022	Low	Low	Unclear	Unclear	Low	Low	High	Unclear	High
Yan et al	2021	Low	Low	Low	Low	Low	Low	Low	Low	Low

Risk of bias (RoB) assessment using Prediction Model Risk of Bias Assessment Tool (PROBAST) where red denotes high risk, green low risk, and yellow unclear risk.

### Radiomics Quality Assessment

RQS ranged from 19.44% to 63.89%. The average RQS across all studies was 36.95% which is consider generally poor based on exploratory studies.^31^ The individual RQS for each study are detailed (see Tables 5 and 6).

**Table 5.** Characteristics of diagnostic radiomic studies of CT paranasal sinuses imaging.

Category	Author	Year	Index test	Reference test	Pathologies	Level of evidence	Radiomics quality score (%)	AUC-ROC	Sensitivity	Specificity	PPV	NPV	Accuracy	Diagnostic utility rating	Other measure of performance
Diagnostic	Baracnovic et al	2022	CT radiomics	Radiologist interpretation and confirmation with positive fungal molds, serum IgE and allergic mucin	Allergic fungal rhinosinusitis versus Nonallergic fungal rhinosinusitis	2	19.44%	0.73	0.71	0.64	N/A	N/A	N/A	Good	N/A
Diagnostic	Costa et al	2023	CT radiomics	Radiologist interpretation	Odontogenic versus nonodontogenic maxillary sinusitis	2	19.44%	N/A	N/A	N/A	N/A	N/A	N/A	Unclear	Statistically significant difference in 3 radiomic parameters
Diagnostic	Guo et al	2023	CT radiomics	Histopathological diagnosis	Nasal polyps versus inverting papilloma	2	41.67%	0.92	0.85	0.99	0.99	N/A	0.92	Excellent	N/A
Diagnostic	Ito et al	2022	CT radiomics	Radiologist interpretation	Odontogenic versus nonodontogenic maxillary sinusitis	2	27.78%	0.76	0.72	0.72	N/A	N/A	0.72	Good	N/A
Diagnostic	Liang et al	2019	CT radiomics	Correlation to clinical variables of T-, N-categories and gross tumor volume	CT and MRI radiomic features in nasopharyngeal carcinoma	4	27.78%	N/A	N/A	N/A	N/A	N/A	N/A	Unclear	76.1% of CT radiomic features significantly correlated with clinical features
Diagnostic	Ogawa et al	2021	CT radiomics	Radiologist interpretation and Histopathological diagnosis	Olfactory neuroblastoma versus sinonasal squamous cell carcinoma	2	41.67%	0.83	0.71	0.96	0.92	0.83	0.86	Very good	N/A
Diagnostic	Zhu et al	2023	CT radiomics	Serum eosinophils and tissue eosinophils, CT Lund-Mackay scores by radiologist	Eosinophilic CRSwNP versus non-eosinophilic CRSwNP	2	63.89%	0.815	0.731	0.81	0.715	0.817	0.776	Excellent	N/A

Diagnostic utility scoring based on AUC-ROC or accuracy with the ratings based on Figure 1. Other outcomes utilized included sensitivity, specificity, PPV, and NPV.

Abbreviations: AUC-ROC, area under the curve receiver operating characteristic; CT, computed tomography; MRI, magnetic resonance imaging; NPV, negative predictive value; PPV, positive predictive value.

**Table 6.** Characteristics of Prognostic Radiomic Studies of CT Paranasal Sinuses Imaging.

Category	Author	Year	Pathology	Prognostic outcomes	Time period	Number of events	Level of evidence	Radiomics quality score (RQS)	C statistic	AUC-ROC	Prognostic utility rating	Calibration slope	Calibration intercept	Other
Prognostication	Intarak et al (a)	2022	Nasopharyngeal carcinoma	Overall survival	3 years	Not recorded	3	41.67%	0.860 ± 0.064	0.886 ± 0.043	Very good	N/A	N/A	
Prognostication	Intarak et al (b)	2022	Nasopharyngeal carcinoma	Progression free survival	3 years	Not recorded	3	41.67%	0.757 ± 0.070	0.818 ± 0.052	Very good	N/A	N/A	
Prognostication	Intarak et al (c)	2022	Nasopharyngeal carcinoma	Distant metastasis free survival	3 years	Not recorded	3	41.67%	0.819 ± 0.055	0.781 ± 0.047	Very good	N/A	N/A	
Prognostication	Yan et al	2021	Nasopharyngeal carcinoma	Progression free survival	7 years	88	3	55.56%	0.873 (95% CI 0.803∼0.943)	0.925	Excellent	0.69	0.053	
Prognostication	Li et al	2022	Chronic Rhinosinusitis	Post endoscopic sinus surgery CT findings	Not recorded	Not recorded	4	30.56%	N/A	N/A	Unclear	N/A	N/A	Misclassification rate of 25.27%

Prognostic utility scoring based on AUC-ROC or accuracy with ratings based on Figure 1. Other outcomes utilized included concordance statistic (C statistic), calibration slope, and calibration intercept.

Abbreviations: AUC-ROC, area under the curve receiver operating characteristic; CT, computed tomography.

### Summary of Diagnostic Studies

There were 7 diagnostic studies included in this review of CT paranasal sinus radiomics^21,22,24,26,28–30^ All 7 utilized CT radiomics as an index test^21,22,24,26–30^, with 1 utilizing MRI radiomics in conjunction^21^. The reference tests were either radiologist interpretation alone (n = 3)^26,28,30^, histopathological diagnosis alone (n = 1)^29^, a combination of both (n = 2)^22,24^, or clinical features such as TNM categories (n = 1)^21^. The pathologies analyzed included odontogenic maxillary sinusitis (n = 2)^26,28^, chronic rhinosinusitis (CRS) with subtypes including allergic fungal rhinosinusitis (AFRS) and eosinophilic CRS with nasal polyps (eCRSwNP) (n = 3)^24,26,30^, inverting papilloma (n = 1)^29^, nasopharyngeal carcinoma (n = 1)^21^, and olfactory neuroblastoma versus sinonasal squamous cell carcinoma (n = 1)^22^. Five studies reported AUC-ROC in the range of 0.73 to 0.92 with diagnostic utility ratings of excellent (n = 2)^29,30^, very good (n = 1)^22^, and good (n = 2)^24,26^. Two studies did not report AUC-ROC or accuracy, with one reporting mean radiomic parameter value with statistically significant differences and one reporting percentage of significant correlation to clinical features^21,28^ (see Table 5).

### Summary of Prognostic Studies

There were 3 studies which analyzed prognostication in paranasal sinuses CT radiomics.^23,25,27^ Two studies used the pathology of nasopharyngeal carcinoma.^23,25^ The prognostic outcomes measured were progression free survival (n = 2)^23,25^, overall survival (n = 1)^25^, and distant metastasis survival (n = 1)^25^. The remaining study utilized CT imaging to predict treatment of CRS post endoscopic sinus surgery.^27^ Two studies reported C statistic and AUC-ROC, with excellent (n = 1)^23^ and very good (n = 1)^25^ diagnostic utility ratings, while the other study used misclassification of disease rate^27^ (see Table 6).

## Discussion

Radiomics as quantitative field of image analysis was first discussed in 2012 by Lambin et al^32^ for the purpose studying intratumour features on medical imaging. Since then, there has been a rapid expansion of radiomics to multiple fields of medicine given the routine use of medical imaging for diagnostics.^33^ As there is routine use of CT imaging in the field of rhinology, radiomics is becoming an increasingly utilized tool, with 3 previous systematic reviews detailing radiomics use in nasopharyngeal carcinoma.^34–36^ This review assesses the use of radiomics in rhinology given the emergence of literature in a variety of rhinological diseases. Radiomics has demonstrated diagnostic accuracy in diseases such as chronic rhinosinusitis including subtypes such as AFRS, odontogenic sinusitis and eosinophilic CRS, nasopharyngeal carcinoma, inverting papillomas, olfactory neuroblastoma and sinonasal squamous cell carcinoma.^21,22,24,26–30^ There are also prognostic benefits with studies utilizing radiomics in nasopharyngeal carcinoma to predict overall survival, progression free survival and distant metastasis free survival due to imaging features of extent or invasiveness that provides prognostics value.^23,25^ Initial studies focused on data localized to single functional areas, that is, maxillary sinuses with user defined radiomic features analyzed which used first order or second order features.^28^ Recent studies exhibit an increasing breadth of complexity and utility of machine learning for radiomic analysis of first-, second-, and higher-order features beyond the capabilities of human alone, for instance, by clustering and identifying eosinophilic chronic rhinosinusitis with nasal polyps and correlating to clinical features.^30^ Studies using increasing complexities of methods in a variety of rhinology diseases are resulting in more opportunities for clinical translation for medical practitioners. Most studies did not use feature selection methods which are useful for minimizing the impact of overfitting in radiomics and improving interpretability, making it challenging to derive meaningful conclusions about their quality. Classification models varied from more simplistic logistic regression and support vector machine used for binary task classification in smaller datasets, to complex unsupervised clustering to identify multiple new groupings.^37,38^ The CV techniques were used to validate the classification models with unseen data. A 10-fold CV was the most common one, as a common method in image analysis, while leave out one CV has value in smaller datasets with comprehensive evaluation although is a time consuming technique.^38^ Overall, when considering feature selection, classification, and CV models there is no single answer for all scenarios and careful consideration of the intended task helps determine which to use. As a result, interpreting data from differing models is challenging, as one is not truly able to compare like for like unless the exact same task and methodology are used. The results of the identified studies show promising results for clinical translation with diagnostic and prognostic performances in the good and above ranges (>0.7) for 70% of studies (n = 7).^22–26,29,30^ And 30% of studies (n = 3)^23,29,30^ even had excellent performances in AUC-ROC with the highest at 0.925.^23^

However, the low to average RQS highlight the inadequate methodological quality of current literature. This review found a low RQS of only 36.95%. This may be related to the exploratory and pilot nature of the radiomics involved, that is, identifying radiomic features in odontogenic maxillary sinusitis may have low clinical utility as most ENT surgeons are taught to identify these features of odontogenic sinusitis on CT. The domains of the RQS which most frequently required improvement included public access to protocols, coding and data, lack of standardization in images’ preprocessing, the low number of prospective studies, lack of interscanner phantom assessment, lack of multitemporal imaging and lack of cost-effectiveness analyses. This assessment remains consistent among multiple fields of medicine with a mean RQS of only 26.1% and specific research areas such as cholangiocarcinoma radiomics ranging between 23% and 39% and neuro-oncology radiomics averaging 37.1%.^33,39,40^ While there are limitations to the RQS, notably with user interpretation, strict adherence to a single ideal radiomic workflow and minimal weightage to clinical translation, currently this remains the most used metric for quality studies.^1^

Limitations of this review included single author study selection as a source of bias, the restriction to the past 5 years for inclusion and the heterogeneous data presented limiting recommendations. This study captured the past 5 years of literature, which was chosen given the dramatic increase in the quantity and quality of radiomic literature.^7^ In the future when more studies are available in this field, subgroup analysis based on pathology would also be beneficial for improved analysis.

A heavily cited issue for radiomics is clinical translation.^40^ While these studies show some promise in prognosticating outcomes in nasopharyngeal carcinoma or aiding radiologist or rhinologist interpretation of CT images, one must ensure that radiomics should be used to help answer clinically relevant questions. Limiting radiomics to identify simple diagnostic features on CT scans which can otherwise be easily interpreted by clinicians, hinders it performing to its full potential. Radiomics is more beneficial when it can identify patterns of disease which require very large datasets and vast experience, or minute and difficult to discern features which are challenging for humans. Diagnostic value may be enhanced with utilizing radiomics for identifying unique and specific features of diseases, and prognostic value with the detection of minute invasion and patterns/characteristics which may proceed the development of disease. Then radiomics can augment current clinical practice, rather than simply replace clinicians as is often questioned in the media and literature with radiomics and AI.

### Implications for Future Research

**Figure 3.**
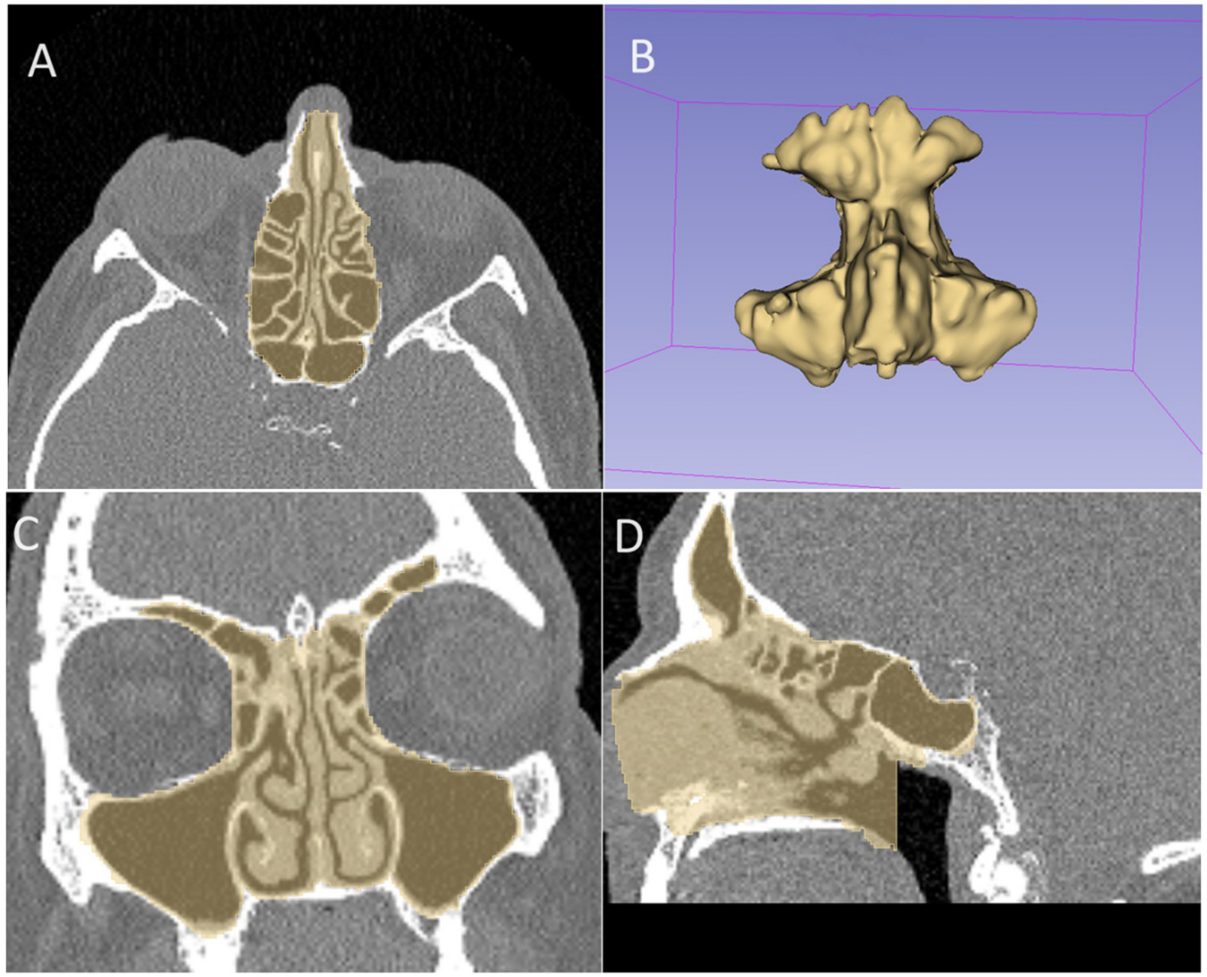
Sample segmentation of the paranasal sinuses for radiomic analysis. Screenshot from 3-dimensional (3D) Slicer (Open Source, http://www.slicer.org) application demonstrating a sample manual segmentation of the paranasal sinuses on computed tomography (CT) imaging, with axial views (A), 3D segmentation of the paranasal sinuses (B), coronal views (C), and sagittal views (D).

From this systematic review, there are potential avenues for further quality radiomic research to encompass more areas of rhinology beyond the most frequent pathology of nasopharyngeal carcinoma. Radiomic data is likely to form the basis of future AI-based analysis with the potential to investigate radiomic phenotypes in Chronic Rhinosinusitis from CT imaging, with the aim of identifying new clusters for clinical classification and optimizing targeted therapies for improved long-term outcomes (see Figure 3).

There is also a comparative predominance of radiomic research in diagnostics and a lack of prognostication studies, with only 2 studies till date limited to nasopharyngeal carcinoma. Currently the most common pathology studied was nasopharyngeal carcinoma, however there is the potential to expand to other tumor subtypes in the paranasal sinuses. To improve the quality of future radiomic research, there is a need for both standardized processes and more transparent and public imaging data and codes.^41^ In 2024, a new tool called METhodological RadiomICs Score “METRICS” was developed via modified Delphi method including an international panel for more flexible methodological assessment scoring to ensure consistency in radiomic studies.^19^ Although in its infancy, this offers promise as a method with mandatory and conditional sections depending upon the methodology used, that is, deep learning, machine learning, etc, and giving importance to the key areas of data acquisition, image segmentation, feature extraction and feature analysis. For improving transparency and availability, there are data registries and open source codes available on GitHub in multiple types of oncologic radiomics research such as lung and brain cancers.^41^ However there is a dearth in the field of rhinology.^41^ Addressing the lack of data and resources, could allow researchers to spend less time on manually intensive data collection and preprocessing, and focus more on delivering solutions for clinical translation in radiomics research.

## Conclusion

Considering the reliance of the rhinology profession on CT of the paranasal sinuses, the use of radiomic data is very limited but growing each year in number and complexity. In order to best harness the ability of radiomics, quality methodologies, and presenting clinically relevant questions are vital. This systematic review highlights the beginnings of clinically relevant research with a potential greatly aid the rhinological surgeon in the future.

## Supplemental Material

sj-docx-1-ajr-10.1177_19458924241304082 - Supplemental material for Radiomics of the Paranasal Sinuses: A Systematic Review of Computer-Assisted Techniques to Assess Computed Tomography Radiological Data Supplemental material, sj-docx-1-ajr-10.1177_19458924241304082 for Radiomics of the Paranasal Sinuses: A Systematic Review of Computer-Assisted Techniques to Assess Computed Tomography Radiological Data by Rhea Darbari Kaul, BMed, Peta-Lee Sacks, MBBS, PhD, Cedric Thiel, MD, Janet Rimmer, MD, Larry Kalish, MBBS (Hons I), Raewyn Gay Campbell, BMed (Hons), Raymond Sacks, MBBCH, Antonio Di Ieva, MD, PhD, and Richard John Harvey, MBBS (Hons I), PhD in American Journal of Rhinology & Allergy
