# Supplementary material for: Radiomics of the Paranasal Sinuses: A Systematic Review of Computer-Assisted Techniques to Assess Computed Tomography Radiological Data
Source: Am J Rhinol Allergy. 2024 Dec 16;39(2):147–58. doi: 10.1177/19458924241304082 (PMC11796290; doi:10.1177/19458924241304082)
Supplement: sj-docx-1-ajr-10.1177_19458924241304082 - Supplemental material for Radiomics of the Paranasal Sinuses: A Systematic Review of Computer-Assisted Techniques to Assess Computed Tomography Radiological Data [file sj-docx-1-ajr-10.1177_19458924241304082.docx]

**Supplementary material**

**Appendix A:**

**Table 1: Patient, intervention, comparison, outcome (PICO) framework.** Table of PICO framework used for this systematic review.

| **Population** | Adults (>18 years) |
| --- | --- |
| **Intervention** | CT paranasal sinus imaging |
| **Comparison** | Radiomics with or without machine learning analysis |
| **Outcomes** | Diagnosis of rhinologic disease or Prognostication of rhinologic disease |
